# Spontaneous Regression of a Symptomatic Intramedullary Spinal Cord Lesion

**DOI:** 10.7759/cureus.7271

**Published:** 2020-03-14

**Authors:** Anthony Mikula, Peter Kalina, Irene Meissner, William E Krauss

**Affiliations:** 1 Neurosurgery, Mayo Clinic, Rochester, USA; 2 Radiology, Mayo Clinic, Rochester, USA; 3 Neurology, Mayo Clinic, Rochester, USA

**Keywords:** intramedullary, spinal cord, spontaneous regression

## Abstract

Intramedullary spinal cord tumors and cavernous malformations are rare lesions that can lead to progressive neurologic deficits, impaired quality of life, and even death. Early diagnosis and surgical resection of spinal cord tumors and cavernous malformations are often quoted as essential to optimizing a patient’s functional outcome. Unfortunately, these are high-risk operations, with many patients having worse neurological deficits after surgery - sometimes permanent.

We present a case of a patient with a cervical intramedullary spinal cord lesion that almost completely resolved spontaneously at short-term follow-up and remained stable at longe-term follow up. Conservative management with careful observation and sequential imaging should be considered in patients with intramedullary spinal cord lesions presenting with acute onset, stable symptoms, especially if the lesion has a hemorrhagic component.

## Introduction

Intramedullary spinal cord tumors are rare, representing only 20%-30% of all primary spinal cord tumors [1]. Most of these tumors are either astrocytomas (60%-70%) or ependymomas (30%-40%) followed distantly by hemangioblastomas [2]. These tumors can lead to progressive severe neurologic deficits, impaired quality of life, and even death. Early diagnosis and surgical intervention have been quoted as essential to optimizing a patient’s functional outcome [3]. However, these are high-risk operations, with 9% to 34% of patients experiencing worsening neurologic deficits following surgery. Only 25%-41% of those patients regain their preoperative clinical status within six months after surgery [4-6].

Intramedullary spinal cord cavernous malformations are also rare lesions that can present acutely with either a stepwise or slow progression [7]. Re-bleed rates of 2% have been reported in the literature, with better outcomes reported for patients who underwent resection versus conservative management, especially if resection is performed within three months of symptoms onset [7]. We present a case of a cervical intramedullary spinal cord lesion that almost completely resolved spontaneously after short-term follow-up.

## Case presentation

History

A 28-year-old man with no significant past medical history presented with neck pain and right arm dysfunction. His symptoms started one month prior to presentation when he awoke with right-sided neck pain and numbness in his right forearm and hand. His symptoms progressed to include right-hand clumsiness and weakness two weeks prior to presentation.

Exam

His exam was notable for 4+/5 weakness throughout his right upper extremity and diffuse numbness across all dermatomes of the right arm and hand. He was hyporeflexic throughout the right upper extremity with the exception of brisk finger flexors. Lower extremity reflexes were symmetrically brisk with non-sustained clonus. He had a positive Babinski on the right and an equivocal plantar response on the left.

Imaging

**Figure 1:**
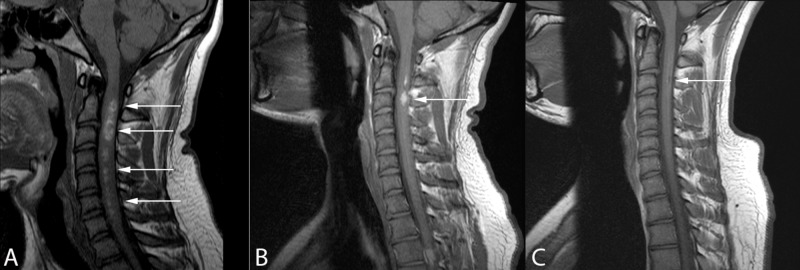
Sagittal T1-weighted MRI at patient presentation (A), three-week follow-up (B), and nine-week follow-up (C) depicting almost complete spontaneous resolution of the cervical intramedullary spinal cord lesion (arrows)

**Figure 2:**
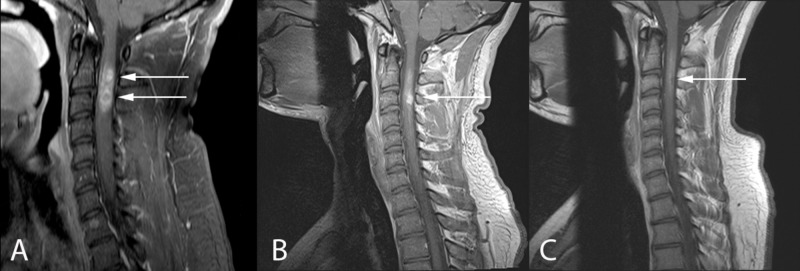
Sagittal T1-weighted MRI with gadolinium at patient presentation (A), three-week follow-up (B), and nine-week follow-up (C) depicting almost complete spontaneous resolution of the cervical intramedullary spinal cord lesion (arrows)

**Figure 3:**
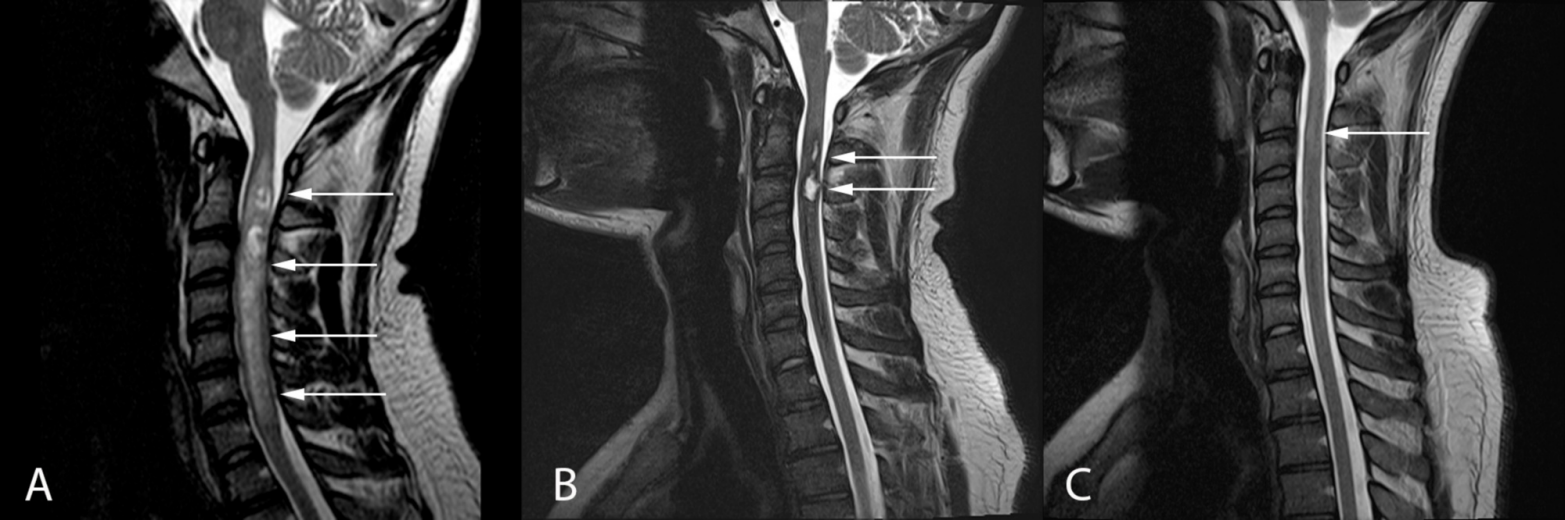
Sagittal T2-weighted MRI at patient presentation (A), three-week follow-up (B), and nine-week follow-up (C) depicting almost complete spontaneous resolution of the cervical intramedullary spinal cord lesion (arrows)

Cervical spine MRI demonstrated an expansile, enhancing intramedullary lesion extending from C2-C3, with associated hemorrhage and edema extending C2-7 (Figures 1A-3A). The lesion was hyperintense on T1- and T2-weighted sequences. It involved the central and dorsal aspect of the cord at C2, with extension into the right posterolateral aspect of the cord between C3 and C7. A thin peripheral rim of T2 hypointensity on the right at C2-3 was compatible with hemosiderin deposition. The most likely differential diagnostic considerations included hemorrhagic ependymoma or astrocytoma, less likely a cavernous malformation.

Subsequent course

A five-day course of prednisone did not improve his symptoms. The tentative plan was to offer the patient a biopsy and possible C2-C7 laminectomy for resection and a C2-T2 posterior instrumented fusion. Follow-up MRI three weeks after initial imaging demonstrated dramatic improvement in the perilesional blood products, along with decreased cord expansion (Figures 1B-3B). The appearance of the lesion was now more suggestive of a cavernous malformation with associated hemorrhage. The decision was made to delay surgery. Repeat MRI six weeks later demonstrated almost complete resolution of the lesion (Figures 1C-3C). This was accompanied by clinical improvement in the patient’s right arm clumsiness and numbness. An additional MRI three months later showed no change and the patient continued to clinically improve.

## Discussion

We present a patient with neck pain and right upper extremity dysfunction who was found to have an enhancing and hemorrhagic intramedullary C2-7 cervical spinal cord lesion. This patient was initially considered for surgical resection entailing a C2-C7 laminectomy followed by a posterior instrumented fusion. The risks of the operation would have been extensive, including worsened neurologic deficits, quadriparesis, ventilator dependence, and even death. However, the sequential repeat MRI demonstrated progressive radiographic improvement with essential resolution of the lesion, as well as associated clinical signs and symptoms.

Few studies have outlined the conservative management and, therefore, the natural history of intramedullary spinal cord lesions, including cavernous malformations. Zhang et al. performed a retrospective study of 85 patients with cavernous malformations, 58 of which underwent surgical resection and 27 who were managed conservatively [8]. Ultimately, the patients who underwent surgery had better clinical outcomes, with 69% having an improvement, 28% remaining unchanged, and 3% having deterioration in neurologic function. In the patients who were managed conservatively, 15% had improvement in their symptoms, 70% remained unchanged, and 15% had a worsening neurologic function. Badhiwala et al. performed a meta-analysis of 632 patients with intramedullary spinal cord cavernous malformations, 90% of which underwent resection and the other 10% were management conservatively [7]. Patients who were managed surgically did better than those who were observed, especially if resection was performed within three months of symptom onset. Of those who were managed conservatively, at last follow-up, 30% had improved, 59% were unchanged, and 11% had worsened symptoms.

Among patients with intramedullary spinal cord tumors, the clinical course and outcomes following surgical resection have been well documented [4,9-11]. In a study by Karikari et al., 102 patients underwent resection of intramedullary spinal cord tumors [11]. At the time of the last follow-up, 14% of patients had improvement in their preoperative symptoms while 18% had a worsening of symptoms. The authors do not comment on the length of symptomatology prior to resection or if repeat imaging was performed preoperatively. Raco et al. reported on 202 patients with intramedullary spinal cord tumors that underwent resection [9]. The duration of clinical symptoms ranged from two months to 20 years with a mean of three years; although they do not comment on whether repeat pre-operative imaging was performed. Postoperatively, 25% of patients with ependymomas improved and 9% of patients worsened. Among those with astrocytomas, 26% noted improvement and 9% had clinical worsening.

## Conclusions

In a patient with an intramedullary spinal cord lesion who is clinically stable without significant symptom progression, it may be reasonable to repeat imaging in four to six weeks to evaluate for spontaneous improvement prior to surgery given the high-risk nature of the operation. This may be especially true for patients who present with acute onset of symptoms with a hemorrhagic lesion on imaging, as the hemorrhagic component can lead to the lesion’s own destruction and may be more likely to resolve spontaneously.

## References

[1] Duong LM, McCarthy BJ, McLendon RE, Dolecek TA, Kruchko C, Douglas LL, Ajani UA (2012). Descriptive epidemiology of malignant and nonmalignant primary spinal cord, spinal meninges, and cauda equina tumors, United States, 2004-2007. Cancer.

[2] Samartzis D, Gillis CC, Shih P, O'Toole JE, Fessler RG (2015). Intramedullary spinal cord tumors: part I—epidemiology, pathophysiology, and diagnosis. Global Spine J.

[3] Samartzis D, Gillis CC, Shih P, O'Toole JE, Fessler RG (2016). Intramedullary spinal cord tumors: Part II—management options and outcomes. Global Spine J.

[4] Garces-Ambrossi GL, McGirt MJ, Mehta VA (2009). Factors associated with progression-free survival and long-term neurological outcome after resection of intramedullary spinal cord tumors: analysis of 101 consecutive cases. J Neurosurg Spine.

[5] Eroes CA, Zausinger S, Kreth FW, Goldbrunner R, Tonn JC (2010). Intramedullary low grade astrocytoma and ependymoma. Surgical results and predicting factors for clinical outcome. Acta Neurochir (Wien).

[6] Cristante L, Herrmann HD (1994). Surgical management of intramedullary spinal cord tumors: functional outcome and sources of morbidity. Neurosurgery.

[7] Badhiwala JH, Farrokhyar F, Alhazzani W (2014). Surgical outcomes and natural history of intramedullary spinal cord cavernous malformations: a single-center series and meta-analysis of individual patient data: clinic article. J Neurosurg Spine.

[8] Zhang L, Yang W, Jia W, Kong D, Yang J, Wang G, Xu Y (2016). Comparison of outcome between surgical and conservative management of symptomatic spinal cord cavernous malformations. Neurosurgery.

[9] Raco A, Esposito V, Lenzi J, Piccirilli M, Delfini R, Cantore G (2005). Long-term follow-up of intramedullary spinal cord tumors: a series of 202 cases. Neurosurgery.

[10] Klekamp J (2013). Treatment of intramedullary tumors: analysis of surgical morbidity and long-term results. J Neurosurg Spine.

[11] Karikari IO, Nimjee SM, Hodges TR (2011). Impact of tumor histology on resectability and neurological outcome in primary intramedullary spinal cord tumors: a single-center experience with 102 patients. Neurosurgery.

